# Sex Differences during Influenza A Virus Infection and Vaccination and Comparison of Cytokine and Antibody Responses between Plasma and Serum Samples

**DOI:** 10.3390/pathogens13060468

**Published:** 2024-06-01

**Authors:** Santosh Dhakal, Brian W. Wolfe, Saurav Pantha, Saranya Vijayakumar

**Affiliations:** Department of Diagnostic Medicine/Pathobiology, College of Veterinary Medicine, Kansas State University, 1800 Denison Avenue, Manhattan, KS 66506, USA; bwwolfe@vet.k-state.edu (B.W.W.); sauravvet@vet.k-state.edu (S.P.); saranya@vet.k-state.edu (S.V.)

**Keywords:** antibody responses, cytokines, influenza vaccine, sex difference

## Abstract

In this study, we evaluated sex differences during infection with mouse-adapted H1N1 and H3N2 influenza A viruses (IAVs) in the C57BL/6J mouse model and compared the cytokine and antibody responses between plasma and serum samples during IAV infection and vaccination. Lethal doses for both H1N1 and H3N2 IAVs were lower for adult females and they suffered with greater morbidity than adult males when infected with sublethal doses. In influenza virus-infected mice, cytokine responses differed between plasma and serum samples. After inactivated influenza virus vaccination and drift variant challenge, adult female mice had greater antibody responses and were better protected. In influenza-vaccinated and challenged mice, binding antibodies were unaffected between paired plasma or serum samples. However, functional antibody assays, including hemagglutination inhibition, microneutralization, and antibody-dependent cellular cytotoxicity assays, were affected by the use of plasma and serum sample types. Our results indicate that careful consideration is required while selecting plasma versus serum samples to measure cytokine and antibody responses during IAV infection and vaccination.

## 1. Introduction

The influenza virus epidemic causes nearly 1 billion infections, up to 5 million severe illnesses, and between 300,000–500,000 deaths every year globally [1]. Different strains of H1N1 and H3N2 influenza A viruses (IAVs) and two lineages of influenza B viruses (IBVs) are responsible for the seasonal human influenza outbreaks. The production of large quantity of pro-inflammatory cytokines is one of several physiological factors playing a role in influenza virus-associated disease severity and deaths [2]. Excessive cytokine production increases the risk of complications such as acute respiratory distress syndrome (ARDS) and multi-organ failure [2].

Vaccine formulations against seasonal influenza contain one strain each of H1N1 and H3N2 IAVs and one or two strains of IBVs. Protection through seasonal influenza vaccination primarily depends on the induction of antibody responses and antibody-mediated virus neutralization [3]. These antibodies mainly target hemagglutinin (HA), the major surface glycoprotein of the influenza virus, and prevent the binding of the virus to the receptors on the respiratory epithelial cells. The antibody responses to seasonal influenza vaccines, however, are strain-specific and the influenza vaccine requires an annual update to protect against frequently evolving virus strains [4].

Various host-associated factors, including biological sex, play significant roles as modifiers of the outcomes of influenza virus pathogenesis and vaccine responses [5,6]. Biological sex refers to the differences between being male or female as determined by the chromosomal makeup (i.e., having XX versus XY chromosome), reproductive organs (i.e., having ovaries versus testes), hormonal differences (i.e., presence of estradiol versus testosterone), and their interactions [5]. Human and mouse model studies have shown that after infection with IAVs adult females suffer from greater morbidity and mortality compared to adult males [7,8,9,10,11,12,13]. However, after vaccination with inactivated influenza virus vaccines, adult females produce greater binding and functional antibody responses in humans and mice and are better protected than adult males in virus-challenge experiments in mouse models [14,15,16,17,18,19,20,21].

In this study, using the C57BL/6J mouse model, we reproduced the findings of sex differences during H1N1 and H3N2 IAV infection and after inactivated influenza virus vaccination. Using the paired plasma and serum samples collected after H1N1 and H3N2 IAV infection, we compared the cytokine responses. Uniquely, we also compared the binding and functional antibody responses between plasma and serum samples collected from influenza virus-vaccinated and challenged mice. Our results suggest that the selection of plasma or serum sample types could affect the measurement of cytokine and functional antibody responses during IAV infection and vaccination.

## 2. Materials and Methods

### 2.1. Animals

Adult (8–10 weeks old) male and female C57BL/6J mice (strain 000664) purchased from Jackson Laboratory were used in this experiment. The animals were housed in the animal biosafety laboratory (ABSL)-2 facility of Kansas State University (KSU). All the animal procedures were approved through the Institutional Animal Care and Use Committee (IACUC) of KSU (approval number 4855).

### 2.2. Viruses

The viruses used in this experiment included: (i) mouse-adapted A/California/04/2009 H1N1 pandemic IAV (referred to as ma2009 H1N1), (ii) a drift variant of mouse-adapted A/California/04/2009 H1N1 containing a K166Q mutation in the HA sequence (referred to as ma2009 H1N1dv), and (iii) mouse-adapted A/Hong Kong/1/1968 H3N2 (referred to as maH3N2). These ma2009 H1N1, ma2009 H1N1dv, and maH3N2 IAVs were kindly provided by Drs. Andrew S. Pekosz and Sabra L. Klein of the Johns Hopkins Bloomberg School of Public Health, Baltimore, Maryland, USA. These viruses have been used in earlier mouse infection and vaccination studies [20,21,22]. From the stock, influenza viruses to be used for infection and vaccination were grown on Madin-Darby Canine Kidney (MDCK) cells after infection with 0.01 multiplicity of infection (MOI).

### 2.3. Influenza Virus Infection and Pathogenesis Study

Adult male and female mice were inoculated either with 10^1^, 10^1.5^, 10^2^, 10^3^, or 10^4^ tissue-culture infectious dose (TCID_50_) of the ma2009 H1N1 or one of the 10^1^, 10^1.5^, 10^2^, 10^3^, or 10^4^ or 10^5^ TCID_50_ of the maH3N2 IAVs. A total of three to five mice per dose per sex were used for the survival studies. The virus was diluted in Dulbecco’s Modified Eagle Medium (DMEM, #11965118, Thermo Fisher Scientific, Waltham, MA, USA) inoculum and administered through the intranasal route under the anesthesia of ketamine (80–100 mg/Kg) and xylazine (5–10 mg/Kg) cocktail. Virus-containing inoculum was administered 15 µL/nare (i.e., in 30 µL total volume). Body mass was recorded before infection and daily after infection until 14-days post-inoculation (dpi). Infected mice were also monitored for clinical signs, including hunched back, and dyspnea. While monitoring animals, those who were in severe distress, moribund, or lost body mass of equal or more than 25% of the baseline body mass were humanely euthanized. Mice that recovered from infection were euthanized at 14-dpi and heparinized plasma samples were collected for antibody measurements. A subset of mice infected with 10^1.5^ TCID_50_ of ma2009 H1N1 and 10^2^ TCID_50_ of maH3N2 IAVs were euthanized at 3-dpi to collect lungs for virus titer measurement and to compare cytokine responses in plasma and serum samples (described in Section 2.7).

### 2.4. Influenza Virus Vaccination and Challenge Study

For vaccine preparation, ma2009 H1N1 IAV was grown on MDCK cells by infecting them with 0.01 MOI. After more than 80% of cells were detached, the supernatant was harvested and clarified by spinning at 500 g for 10 min and at 4 °C temperature. IAVs were inactivated using beta-propiolactone (#AAB2319703, Thermo Fisher Scientific, Waltham, MA, USA), and then stored at −80 °C until ultracentrifugation. For ultracentrifugation, the stored supernatant was thawed and then centrifuged at 28,000 rpm for 1 h at 4 °C using Sorvall WX+ Ultracentrifuge (Thermo Fisher Scientific, Waltham, MA, USA). Protein estimation was carried out using BCA protein assay kit (#PI23227, Thermo Fisher Scientific, Waltham, MA, USA). Animals were vaccinated with 20 µg of inactivated ma2009 H1N1 vaccine diluted in 40 µL of sterile 1x phosphate-buffered saline (PBS) through an intramuscular route in the right thigh muscle. Mice were boosted after 3 weeks. At 35-days post-vaccination (dpv), heparinized plasma samples were collected for antibody measurement. At 42-dpv, vaccinated mice were challenged with a 10^5^ TCID_50_ of the ma2009 H1N1dv virus. Body mass was recorded before infection and daily after infection until 14-days post-challenge (dpc). For comparison of antibody responses between plasma and serum samples, samples collected from a subset of vaccinated and challenged mice euthanized at 14-dpc were used (described in Section 2.8, Section 2.9,Section 2.10, Section 2.11 and Section 2.12).

### 2.5. Virus Titration in Lung Homogenates

For virus titration, lungs were collected from a subset of mice infected with 10^1.5^ TCID_50_ of ma2009 H1N1 or 10^2^ TCID_50_ of maH3N2 IAVs at 3-dpi (described in Section 2.3). The lungs were weighed, and DMEM was added based on the tissue weight at a rate of 1000 µL per 250 mg. Lung tissues were homogenized (three times, 10 s each on high) using the Fisherbrand 850 homogenizer and sterile disposable plastic generator probes (#15340169 and #15340176, Thermo Fisher Scientific, Waltham, MA, USA). Samples were maintained on ice throughout the procedure. Homogenates were centrifuged at 4000 rpm for 20 min at 4 °C and supernatants were aliquoted and stored at −80 °C until being used for TCID_50_ assay. For the TCID_50_ assay, lung homogenates were 10-fold serially diluted in a serum-free infection medium comprised of DMEM with penicillin–streptomycin antibiotics (#15140122, Thermo Fisher Scientific, Waltham, MA, USA) and L-glutamine (#25030081, Thermo Fisher Scientific, Waltham, MA, USA) and then transferred in six replicates in 96-well cell culture plates (TPP, Bubendorf, Switzerland) confluent with MDCK cells. Plates were incubated at 32 °C for 6 days, fixed with 4% formaldehyde solution, and stained with naphthol blue-black solution. Virus titers were calculated using the Reed and Muench method [23].

### 2.6. Collection of Plasma and Serum Samples

Plasma and serum samples were used to compare cytokine responses after influenza virus infection (described in Section 2.3) or to compare antibody responses after influenza virus vaccination and challenge (described in Section 2.4). For the comparison of cytokine responses, a subset of mice infected with 10^1.5^ TCID_50_ of ma2009 H1N1 or 10^2^ TCID_50_ of maH3N2 were euthanized at 3-dpi. For the comparison of antibody responses, a subset of mice vaccinated with ma2009 H1N1 inactivated vaccine twice at the 3-week interval and challenged with ma2009 H1N1dv at 42-dpv were euthanized at 14-dpc. On the day of euthanization, blood samples were collected through retroorbital bleeding under ketamine and xylazine anesthesia. Plasma samples were obtained by collecting blood in 1.5 mL microcentrifuge tubes containing 50 µL of either heparin sodium (NDC 63739-931-14) or 0.5 M ethylenediaminetetraacetic acid (EDTA, #BP2482100, Thermo Fisher Scientific, Waltham, MA, USA) solutions. After the addition of blood to heparin and EDTA-containing tubes, they were gently mixed, and tubes for plasma separation were immediately transferred into ice. For serum separation, blood samples were directly collected in 1.5 mL microcentrifuge tubes and maintained at room temperature until being centrifuged together. For plasma and serum separation, blood samples were centrifuged at 2000 rpm for 20 min at 4 °C, and the supernatant was harvested and stored at −80 °C until processing for cytokine or antibody assays. The duration between blood sample collection and plasma or serum separation was 1 to 5 h. Hereafter, plasma samples collected in heparin and EDTA are referred to as Plasma-H and Plasma-E, respectively.

### 2.7. Measurement of Cytokines

Cytokines in plasma and serum samples were measured using ProcartaPlex mouse Th1/Th2 cytokine panel, 11plex (#EPX11020820901, Thermo Fisher Scientific, Waltham, MA, USA) as per the manufacturer’s instructions. The cytokines measured were GM-CSF, INF-γ, IL-1β, IL-2, IL-4, IL-5, IL-6, IL-12p70, IL-13, IL-18, and TNF-α. Standards were run in duplicates while test samples were processed in singlicate, owing to the limitation of sample volume. Plate reading was carried out using Luminex xMAP technology (Austin, TX, USA). For analysis, raw data were exported from the xPonent software version 4.2 and imported into the ProcartaPlex analysis application (Thermo Fisher Scientific, Waltham, MA, USA). Cytokine concentrations (pg/mL) were calculated for each sample according to a five-parameter line fitted to the seven standards provided in the kit. If cytokine concentration was low and could not be determined in any samples, a value of half of the lowest detected concentration for that particular cytokine was used to enable statistical analysis. To compare the net median fluorescence intensity (Net-MFI) values, any negative MFI observations were set to zero.

### 2.8. Antibody Measurement by Enzyme-Linked Immunosorbent Assays (ELISAs)

Immunoglobulin M (IgM), IgG, IgG1, IgG2a, and IgG2c antibody levels were measured on plasma and serum samples by ELISAs as described earlier [21]. Briefly, 96-well ELISA plates (#655081, Greiner Bio-One, Monroe, NC, USA) were coated with 50 µL/well of ma2009 H1N1 or maH3N2 whole virus proteins diluted at 2 µg/mL concentration on sodium carbonate and sodium bicarbonate buffer. After overnight incubation at 4 °C, plates were blocked with 10% skim milk solution, followed by the addition of two-fold serially diluted plasma or serum samples. After 1 h incubation at 37 °C with samples, plates were washed, and horse-radish peroxidase (HRP)-conjugated secondary IgM (#626820, Invitrogen, Waltham, MA, USA), IgG (#PI32430, Invitrogen, Waltham, MA, USA), IgG1 (#PA174421, Invitrogen, Waltham, MA, USA), IgG2a (#A10685, Invitrogen, Waltham, MA, USA), or IgG2c (#56970, Cell Signaling Technology, Danvers, MA, USA) antibodies were added. The reaction was developed using the TMB substrate reagent set (#555214, BD BIosceinces, San Jose, CA, USA) and stopped after 15–20 min using 1N hydrochloric acid. Optical density (OD) values were measured at 450 nm wavelength using the ELISA plate reader (Biotek, Winooski, VT, USA) and the endpoint titer was calculated as the highest dilution of the plasma or serum samples having OD values greater than three times the average OD of the negative controls.

### 2.9. Antibody Avidity Assay

Antibody avidity assay was performed using ammonium thiocyanate solution as described earlier [23]. The antibody avidity assay procedure was similar to the antibody ELISA protocol described above with a few changes. First, plasma or serum samples were used only at 1:50 dilution. Second, after incubation with samples and before adding a secondary IgG-HRP antibody, plates were incubated with different molarities (i.e., 1, 2, 4, and 8 molar) of ammonium thiocyanate solution for 15 min. The avidity index was calculated as the ratio of the OD value of ammonium thiocyanate treated wells to the corresponding OD from control wells for the respective samples.

### 2.10. Microneutralization Assay

Virus-neutralizing antibody (nAb) titers on plasma and serum samples were determined using the microneutralization assay [20,23]. Briefly, plasma or serum samples were two-fold serially diluted starting with 1:20 dilution, incubated with 100 TCID_50_ of ma2009 H1N1 or maH3N2 IAVs, and then transferred to 96-well cell culture plates confluent with MDCK cells. Plates were incubated at 32 °C, 5% CO_2_ incubator for 6 days followed by fixation with 4% formaldehyde and staining with naphthol blue-black solution. nAb titer was determined as the highest dilution where virus growth was inhibited.

### 2.11. Hemagglutination Inhibition (HI) Assay

Hemagglutination inhibition (HI) antibody assay was performed as described earlier [16]. Briefly, plasma or serum samples were two-fold serially diluted starting at 1:10 dilution and incubated with 4 HA units of ma2009 H1N1 IAV, followed by the addition of 1% turkey red blood cells (RBCs, #ITKRBC5P, Innovative Research, Novi, MI, USA). The HI titer was determined as the highest dilution where virus agglutination was inhibited as depicted by precipitation of RBCs at the bottom of the well.

### 2.12. Antibody-Dependent Cellular Cytotoxicity (ADCC) Reporter Assay

ADCC assay was performed using the mouse FcγRIV ADCC reporter bioassay kit (#M1211, Promega, Madison, WI, USA) [23]. Briefly, white-bottom 96-well cell culture plates were seeded with MDCK cells (20,000/well), and after 24 h incubation (in a 37 °C, 5% CO_2_ incubator) infected with 3 MOI of ma2009 H1N1 IAV to prepare target cells. After another 24 h incubation, 10-fold serially diluted plasma or serum samples were added together with ADCC bioassay effector cells (75,000/well). After 6 h of incubation, Bio-Glo luciferase reagent was added, and luminescence was recorded on FLx800 (Biotek, Winooski, VT, USA). The area under the curve values were determined using GraphPad Prism 10.2.3 software.

### 2.13. Statistical Analysis

After the ma2009 H1N1 and maH3N2 IAVs pathogenesis study, simple survival analysis was performed using the Kaplan–Meier method and mouse lethal dose (LD_50_) was calculated for males and females against both viruses using a dose-response curve and nonlinear regression model. Antibody responses and virus titers in pathogenesis studies between males and females were compared using the Mann–Whitney test. Morbidity data were compared using two-way repeated measures analysis of variance (ANOVA) followed by Tukey’s multiple comparisons. Cytokine and antibody responses between Plasma-H, Plasma-E, and serum samples were compared using one-way ANOVA followed by Tukey’s multiple comparisons tests. Data were analyzed on GraphPad Prism 10 and *p* < 0.05 was considered a significant difference.

## 3. Results

### 3.1. Lethal Doses for Both H1N1 and H3N2 IAVs Are Lower for Adult Females Than for Male Mice

**Figure 1:**
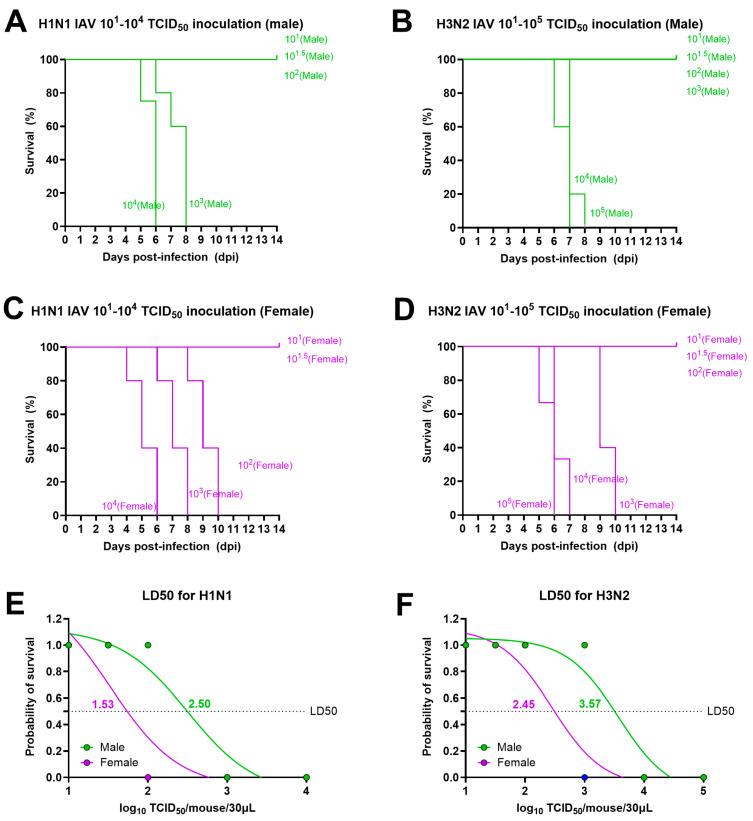
**Lethal doses for H1N1 and H3N2 IAVs for adult male and female mice.** Adult (8–10 weeks old) male and female mice (n = 3–5/sex/group) were infected with 10^1^–10^4^ TCID_50_ of H1N1 and 10^1^–10^5^ TCID_50_ of H3N2 IAVs. Body mass was measured up to 14-dpi, and mice that lost 25% or more body mass from the baseline were humanely euthanized. Simple survival analysis (Kaplan–Meier) for (**A**,**C**) H1N1 and (**B**,**D**) H3N2 IAVs are shown for males (**A**,**B**) and females (**C**,**D**). Determination of 50% mouse lethal dose (LD50) for (**E**) H1N1 and (**F**) H3N2 IAVs are shown using the dose-response curve and nonlinear regression analysis.

Adult (8–10 weeks old) male and female C57BL/6 mice were infected with either 10^1^, 10^1.5^, 10^2^, 10^3^, or 10^4^ TCID_50_ of H1N1 IAV or one of the 10^1^, 10^1.5^, 10^2^, 10^3^, 10^4^, or 10^5^ TCID_50_ doses of H3N2 IAV. Change in body mass was measured in infected mice for a period of 14-dpi and if they lost 25% or more body mass from the baseline, they were humanely euthanized. After infection with H1N1 IAV, all male mice survived and recovered up to a dose of 10^2^ TCID_50_ while females showed survival and recovery only up to 10^1.5^ TCID_50_ dose (Figure 1A,C). Likewise, after infection with the H3N2 virus, all infected males survived and recovered up to a dose of 10^3^ TCID_50_ while females showed survival and recovery only up to 10^2^ TCID_50_ doses (Figure 1B,D). Mice infected with other doses of H1N1 lost more than 25% body mass from the baseline and were humanely euthanized between 4- and 10-dpi. Likewise, mice infected with other doses of H3N2 IAVs lost more than 25% body mass from the baseline and were humanely euthanized between 5- and 10-dpi (Figure 1A–D). Subsequently, LD50s for both viruses were calculated using the dose-response curve and nonlinear regression analysis, and they were lower for females compared with the male mice for both IAVs. The LD50 for the H1N1 virus was 10^1.53^ TCID_50_ for females and 10^2.5^ TCID_50_ for males (Figure 1E). Likewise, LD50 for the H3N2 virus was 10^2.45^ TCID_50_ for females and 10^3.57^ TCID_50_ for males (Figure 1F).

### 3.2. After Infection with Sublethal Doses of Both H1N1 and H3N2 IAVs, Adult Females Suffer from More Severe Disease Than Male Mice

**Figure 2:**
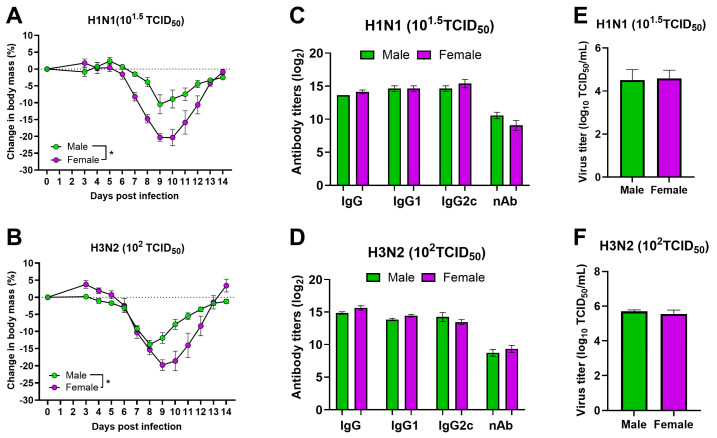
**Morbidity, antibody responses, and lung virus titers after sublethal dose infection with H1N1 and H3N2 IAVs.** Adult (8–10 weeks old) male and female mice were infected either with 10^1.5^ TCID_50_ of H1N1 IAV or 10^2^ TCID_50_ of H3N2 IAV. Percentage changes in body mass up to 14-dpi were measured in mice infected with (**A**) H1N1 and (**B**) H3N2 IAVs. IgG, IgG1, and IgG2c antibodies by ELISAs and nAb responses by microneutralization assays were measured in recovered mice at 14-dpi against (**C**) H1N1 and (**D**) H3N2 IAVs. A subset of mice infected with (**E**) H1N1 and (**F**) H3N2 IAVs were euthanized at 3-dpi, lungs were harvested, and infectious virus titers were determined in lung homogenate. Data represent mean ± standard error of means (SE) of 4–5 animals/group. Changes in body mass were compared using two-way repeated measures ANOVA followed by Tukey’s multiple comparisons test. Antibody and virus titers were compared using the Mann–Whitney test. Asterisk (*) refers to significant differences at *p* < 0.05.

After infection with different doses of H1N1 and H3N2 IAVs, 10^1.5^ TCID_50_ of H1N1 and 10^2^ TCID_50_ of H3N2 IAVs were the highest doses in which all mice survived and recovered in both male and female groups (Figure 1A,B). Thus, we compared the changes in body mass between males and females after infection with these sublethal doses. When infected with 10^1.5^ TCID_50_ of H1N1 and 10^2^ TCID_50_ of H3N2 IAVs, female mice lost significantly greater body mass compared to males (Figure 2A,B). To determine if the severe disease in female mice was associated with their inability to produce antibodies, we measured IgG, IgG1, IgG2c, and neutralizing antibody responses in heparinized plasma samples collected after recovery, i.e., at 14-dpi. IgG, IgG1, and IgG2c antibody titers measured by ELISAs on plasma samples, and virus nAb titers measured by microneutralization assay at 14-dpi were comparable between adult male and female mice after infection with both H1N1 and H3N2 IAVs (Figure 2C,D). To determine if severe disease in females was associated with greater virus replication in the lungs, we euthanized a subset of male and female mice infected either with 10^1.5^ TCID_50_ of H1N1 or 10^2^ TCID_50_ of H3N2 IAVs at 3-dpi. Lungs were collected at 3-dpi, homogenized, and used to measure infectious virus titers by infecting MDCK cells. The pulmonary virus titers measured by TCID_50_ assay in lung homogenates collected at 3-dpi were comparable between adult male and female mice after infection with both H1N1 and H3N2 IAVs (Figure 2E,F).

### 3.3. Cytokine Responses between Plasma and Serum Samples Differ during Infection with Sublethal Doses of H1N1 and H3N2 IAVs

**Figure 3:**
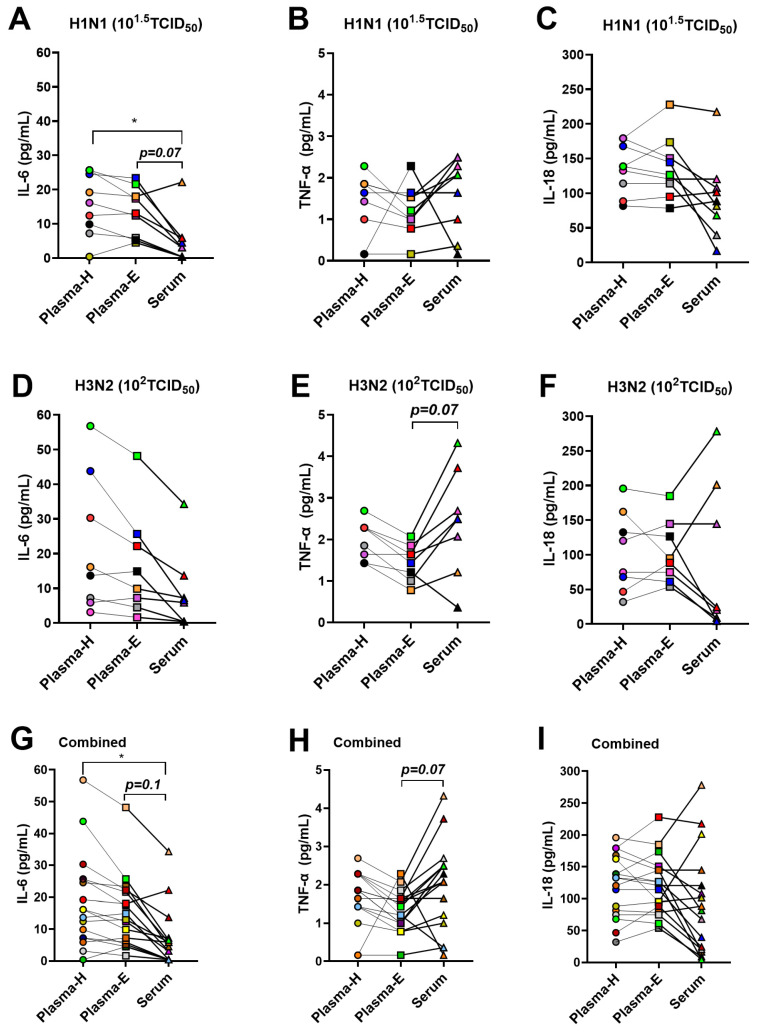
**Comparison of cytokine concentrations between plasma and serum samples.** Adult (8–10 weeks old) male and female mice were infected either with 10^1.5^ TCID_50_ of H1N1 or 10^2^ TCID_50_ of H3N2 IAVs. Mice were euthanized at 3-dpi and blood samples were collected from each mouse to obtain Plasma-H, Plasma-E, and serum samples for the measurement of cytokine responses. Concentrations of (**A**,**D**,**G**) IL-6; (**B**,**E**,**H**) TNF-α, and (**C**,**F**,**I**) IL-18 cytokines are presented. The same color indicates Plasma-H, Plasma-E, and serum samples from the same animal. Data of 8–17 animals are shown as symbols and lines and statistical comparison was performed using one-way ANOVA followed by Tukey’s multiple comparisons test. Asterisk (*) refers to a significant difference at *p* < 0.05.

As mentioned above, a subset of mice infected with the sublethal doses (i.e., 10^1.5^ TCID_50_ of H1N1 and 10^2^ TCID_50_ of H3N2 IAVs) were euthanized at 3-dpi. Paired Plasma-H, Plasma-E, and serum samples were collected from each mouse to compare cytokine responses at 3-dpi. We measured 11 cytokines using the ProcartaPlex Mouse Th1/Th2 cytokine panel. Out of 11 cytokines, only 3, i.e., IL-6, TNF-α, and IL-18, were above detection limits in most of the samples in the systemic circulation. For comparison between plasma and serum samples, data of males and females were combined. During H1N1 IAV infection (Figure 3A–C), IL-6 measurement was significantly greater in Plasma-H, while it showed a higher trend (*p* = 0.07) in Plasma-E, compared to serum samples collected from the respective mice (Figure 3A). TNF-α concentration appeared to be reduced in Plasma-E compared with Plasma-H and serum samples for most of the animals, except for the one mouse which had higher levels in Plasma-E than in Plasma-H and serum samples for unknown reasons (Figure 3B). The concentration of IL-18 was lower in serum samples compared to the Plasma-H and Plasma-E samples in many of the mice (Figure 3C), but the difference was not statistically significant. During H3N2 IAV infection (Figure 3D–F), like the observation after H1N1 IAV infection, the concentration of IL-6 was highest in Plasma-H, medium in Plasma-E, and lowest in serum samples. However, due to the larger variation, there was no statistically significant difference observed (Figure 3D). Consistently, the TNF-α level was lowest in Plasma-E samples and serum samples had a higher trend (*p* = 0.07) of TNF-α measurement than in Plasma-E (Figure 3E). IL-18 levels were lower in serum samples of five (out of eight) mice while three of them had higher concentrations than that in Plasma-H and Plasma-E samples, and data had no statistically significant difference (Figure 3F). We also combined the data of H1N1 and H3N2-infected mice (Figure 3G–I). After combining the data, IL-6 levels in Plasma-H were significantly higher than those in serum samples while Plasma-E had a higher trend (*p* = 0.1) than that of serum samples (Figure 3G). Serum samples had a higher trend (*p* = 0.06) of TNF-α measurement compared to Plasma-E (Figure 3H), while IL-18 levels were comparable between plasma and serum samples (Figure 3I).

For the other cytokines that had concentrations below the detection limits in most of the mice, Net-MFIs were compared (Figure S1A–K). Data from H1N1 and H3N2 infected mice were combined for the Net-MFI analyses. The differences observed between plasma and serum samples for IL-6, TNF-α, and IL-18 cytokines when their concentrations were compared (Figure 3), were consistent even when Net-MFIs were compared. Further, there were significant differences observed in Net-MFIs of different cytokines, including IL-2, IL-4, and IL13, when paired plasma and serum samples were compared (Figure S1).

### 3.4. Vaccinated Adult Female Mice Have a Higher Antibody Response and Are Better Protected Than Male Mice

**Figure 4:**
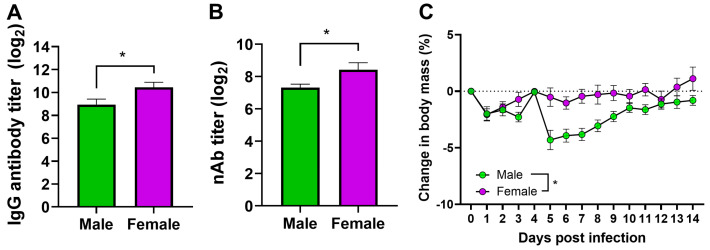
**Vaccinated female mice have higher antibody responses and are better protected following drift H1N1 IAV challenge.** Adult male and female (8–10 weeks old) mice were vaccinated with the 2009 pandemic H1N1 IAV vaccine twice at a 3-week interval. At 35-dpv, heparinized plasma samples were collected, and (**A**) IgG antibody was measured using ELISA and (**B**) virus-neutralizing antibody (nAb) titers were measured by microneutralization assay. (**C**) At 42-dpv, vaccinated mice were challenged with the drift variant of H1N1 IAV, and percentage change in body mass was compared throughout 14-dpc. Data represent mean ± standard error of mean (SEM) of 10 mice/sex. Antibody data were analyzed with an unpaired *T*-test, and percentage change in body mass was compared using two-way repeated measures ANOVA followed by Tukey’s multiple comparisons test. An asterisk (*) refers to a statistically significant difference at *p* < 0.05.

Adult male and female mice were vaccinated with inactivated H1N1 vaccine. At 35-dpv, heparinized plasma samples were collected, and the IgG and virus-neutralizing antibody titers were measured by ELISA and microneutralization assay, respectively. Both the IgG and neutralizing antibody responses were significantly greater in female mice compared to males (Figure 4A,B). At 42-dpv, vaccinated mice were challenged with 10^5^ TCID_50_ of a drift variant of the H1N1 virus. After the virus challenge, body mass was measured daily. Adult females were better protected than adult males after the drift variant virus challenge as they lost significantly less body mass compared to males (Figure 4C).

### 3.5. ELISA-Based Antibody Responses Are Comparable between Plasma and Serum Samples in Vaccinated and Challenged Mice

**Figure 5:**
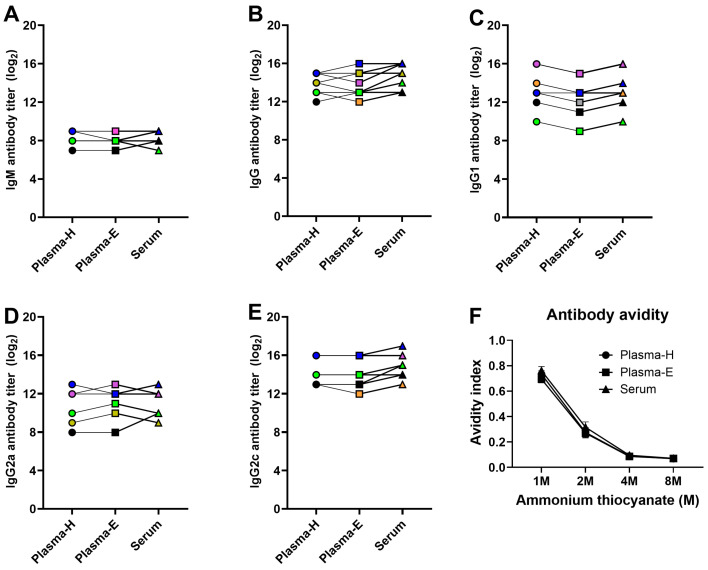
**Comparison of ELISA-based antibody assays between plasma and serum samples.** Adult (8–10 weeks old) male and female mice were vaccinated with inactivated 2009 pandemic H1N1 vaccine at a 3-week interval. Vaccinated animals were challenged with a drift variant of the H1N1 virus at 42-dpv. Mice were euthanized at 14-dpc, and paired plasma and serum samples were collected from each mouse to measure: (**A**) IgM; (**B**) IgG; (**C**) IgG1; (**D**) IgG2a; and (**E**) IgG2c antibodies. Likewise, (**F**) antibody avidity was also measured after treatment of plasma and serum samples with different concentrations of ammonium thiocyanate. Data of 7–8 animals are shown as symbols and lines, and statistical comparison was performed using one-way ANOVA followed by Tukey’s multiple comparisons test. The same color indicates Plasma-H, Plasma-E, and serum samples from the same animal.

To compare antibody responses based on sample type, paired Plasma-H, Plasma-E, and serum samples were collected from vaccinated and challenged animals at 14-dpc. ELISAs were used to measure IgM, IgG, IgG1, IgG2a, and IgG2c antibody levels in plasma and serum samples. Data from male and female mice were combined for plasma and serum antibody comparison. As expected, the level of IgM antibody at 14-dpc was lower than other class-switched antibody isotypes (Figure 5). In addition, the IgM, IgG, IgG1, IgG2a, and IgG2c antibody titers in vaccinated and challenged animals at 14-dpc were similar between Plasma-H, Plasma-E, and serum samples (Figure 5A–E). We then measured antibody avidity by treating plasma and serum samples with different concentrations of ammonium thiocyanate. The avidity indexes at different molarities of ammonium thiocyanate were also comparable between plasma-H, plasma-E, and serum (Figure 5F).

### 3.6. Functional Antibody Assays Are Affected by Plasma or Serum Sample Types

**Figure 6:**
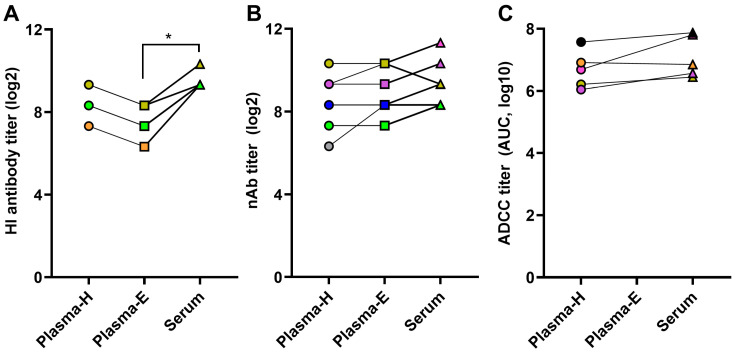
**Comparison of functional antibody assays between plasma and serum samples.** Adult (8–10 weeks old) male and female mice were vaccinated with inactivated 2009 pandemic H1N1 vaccine at a 3-week interval. Vaccinated animals were challenged with a drift variant of the H1N1 virus at 42-dpv. Mice were euthanized at 14-dpc, and plasma and serum samples were collected from each mouse to measure: (**A**) hemagglutination inhibition (HI); (**B**) virus-neutralizing antibody (nAb); and (**C**) antibody-dependent cellular cytotoxicity (ADCC) antibodies. Data of 5–8 animals are shown as symbols and lines, and statistical comparison was performed using one-way ANOVA followed by Tukey’s multiple comparisons test in figures (**A**,**B**) and unpaired *T*-test in figure (**C**). The same color indicates Plasma-H, Plasma-E, and serum samples from the same animal. An asterisk (*) refers to a statistically significant difference at *p* < 0.05.

To compare functional influenza virus-specific antibody responses between sample types, Plasma-H, Plasma-E, and serum samples collected from vaccinated and challenged animals at 14-dpc were used. Plasma and serum samples were subjected to measure functional HI, nAb, and ADCC antibody titers. HI antibody titers were similar between Plasma-H and serum samples. However, Plasma-E had significantly lower HI antibody titers compared to serum samples (Figure 6A). Though serum samples had higher average nAb titers, there was no statistical difference between Plasma-H, Plasma-E, and serum samples (Figure 6B). However, an issue was observed consistently with Plasma-E while performing nAb assay. Naphthol blue-black stain was not observed in initial dilutions (i.e., the lower dilutions) of Plasma-E samples (Figure S2A). As the Plasma-E dilution increased, staining was visible. The ADCC response was also comparable between plasma-H and serum samples (Figure 6C). With Plasma-E samples, however, ADCC antibody titers could not be determined as the luminescence readouts were obscured at lower dilutions (Figure S2B). These data indicate that plasma samples, especially Plasma-E can create issues with functional antibody assays.

## 4. Discussion

Using the C57BL/6J mouse model, we showed that the lethal doses of A/California/04/2009 H1N1 and A/Hong Kong/1/1968 H3N2 IAVs are lower for adult females than adult male mice. Adult females also suffered from severe morbidity (i.e., body mass loss) during sublethal dose infections with both IAV subtypes. These findings replicate previous studies which showed that after infection with different strains of H1N1 and H3N2 IAVs, adult female C57BL6/CR mice from Charles River Laboratory suffer from greater morbidity and mortality, and require lower lethal doses [8,9,10,24]. The greater disease severity in adult female mice was not associated with higher virus replication in the lungs or inability to produce antibody responses. This is also consistent with prior studies which showed that differences in the production of proinflammatory cytokines and chemokines in the lungs and pulmonary pathology rather than pulmonary virus titers were associated with disease severity in females [8,10].

After influenza vaccination and challenge, adult female C57BL/6J mice produced higher antibody responses and were better protected than male mice. This is also consistent with our previous studies in which we showed that vaccinated adult females had higher antibody responses to the influenza vaccine and were better protected after virus challenge due to greater germinal center (GC) responses mediated by estradiol, including higher numbers of GC B cells and plasmablasts and greater somatic hypermutation frequencies of GC B cells [20,21]. Our current study used 25% of body mass loss from the baseline after IAV infection as the criteria for humane endpoint compared to 30% of body mass loss from the baseline used in previous studies [20,21,24].

Blood collection techniques, including the use of anticoagulants, can affect cytokine measurements. In our study, the concentrations, or MFIs, of different cytokines, including IL-6, TNF-α, and IL-4, varied between plasma and serum samples collected from the same animals. For example, the concentration as well as the Net-MFIs of IL-6 cytokine was highest in Plasma-H, medium in Plasma-E, and lowest in serum samples. Prior studies have indicated that more differences in cytokine measurements were observed with heparin plasma than with EDTA plasma and levels of several cytokines in healthy human subjects were elevated in heparin plasma [25,26]. While the exact mechanism for the differences is not clear, depending on the contact time, heparin might likely stimulate blood cells such as monocytes and macrophages, resulting in the secretion of pro-inflammatory cytokines [27]. Plasma-E also had higher concentrations or MFIs of cytokines like IL-6, IL-4, and IL-13 while lower MFIs of TNF-α compared to serum samples of the same animals. Due to EDTA being a calcium-chelating agent, depending on the concentration and duration between blood collection to plasma separation, it might also have an indirect effect on cytokine responses by affecting the viability and activation of cells as it quickens apoptosis and necrosis of cells [28,29,30]. Prior studies showed that serum and plasma cytokines are poorly correlated with each other and are differentially affected by temperature and processing delays [25,31]. In our study, the duration of blood collection to plasma separation varied between 1 to 5 h for different animals, and analyzing the effect of time and different concentrations of heparin or EDTA was beyond the scope of this study.

We also compared binding (i.e., as determined by ELISA-based assays) antibodies and functional antibodies (i.e., HI, nAb, and ADCC-mediating antibodies) between plasma and serum samples collected from mice vaccinated with inactivated influenza vaccine and challenged with a drift variant virus. It was apparent that the ELISA-based antibody responses, such as measurement of virus-specific IgG, IgG1, IgG2a, IgG2c, and IgM antibody responses, and antibody avidity assays were unaffected by plasma and serum sample types. Plasma-H and serum samples had comparable functional antibody responses, whether it be the HI, nAb, or ADCC antibody titers. Defang et al. also showed that heparin plasma samples had a high degree of agreement and correlation with serum samples in HI antibody titers in humans [32]. Plasma-E, however, had an HI antibody titer significantly lower than the paired serum samples. Plasma-E also had issues in the microneutralization and ADCC assays at lower dilutions that affected data interpretation. Since EDTA is a mild cell detachment agent and plays a role in facilitating cellular apoptosis and necrosis, the presence of a higher concentration of EDTA at lower plasma dilutions is probably responsible for the interferences observed in functional antibody assays [33,34].

In this experiment, we used only one concentration of heparin and EDTA and could not determine the effects of different concentrations, contact time with blood, and storage temperatures, all of which could have different impacts on cytokine and antibody responses. However, these findings suggest that the choice of anticoagulant could affect systemic cytokine analysis and antibody assays during influenza virus pathogenesis and vaccine studies.

## 5. Conclusions

Biological sex impacts influenza virus pathogenesis and vaccine responses. Hence, efforts to better understand the underlying mechanisms should be continued in order to develop optimized therapeutics and vaccines. The measurement of cytokine and antibody responses after influenza infection and vaccination are affected by the use of plasma or serum sample types. Hence, careful consideration is required while selecting plasma versus serum samples to measure cytokine and antibody responses during IAV infection and vaccination.

## Data Availability

Data presented in this study will be available upon reasonable request from the corresponding author.

## Supplementary Materials

The following supporting information can be downloaded at: https://www.mdpi.com/article/10.3390/pathogens13060468/s1, Figure S1: Comparison of cytokine responses (median fluorescent intensities, MFIs) between plasma and serum samples; and Figure S2: Issues observed with Plasma-E in functional antibody assays.
