# Supplementary material for: Sex Differences during Influenza A Virus Infection and Vaccination and Comparison of Cytokine and Antibody Responses between Plasma and Serum Samples
Source: Pathogens. 2024 Jun 1;13(6):468. doi: 10.3390/pathogens13060468 (PMC11206404; doi:10.3390/pathogens13060468)
Supplement: Supplementary file 1 [file pathogens-13-00468-s001.zip › pathogens-3012981-supplementary.pdf]

pathogens

MDPI

Article

Sex Differences during Influenza A Virus Infection and Vaccination and Comparison of Cytokine and Antibody Responses between Plasma and Serum Samples

Santosh Dhakal *, Brian W. Wolfe, Saurav Pantha and Saranya Vijayakumar

Department of Diagnostic Medicine/Pathobiology, College of Veterinary Medicine, Kansas State University, 1800 Denison Avenue, Manhattan, KS 66506, USA; bwwolfe@vet.k-state.edu (B.W.W.); sauravvet@vet.k-state.edu (S.P.); saranya@vet.k-state.edu (S.V.)

* Correspondence: sdhakal3@vet.k-state.edu

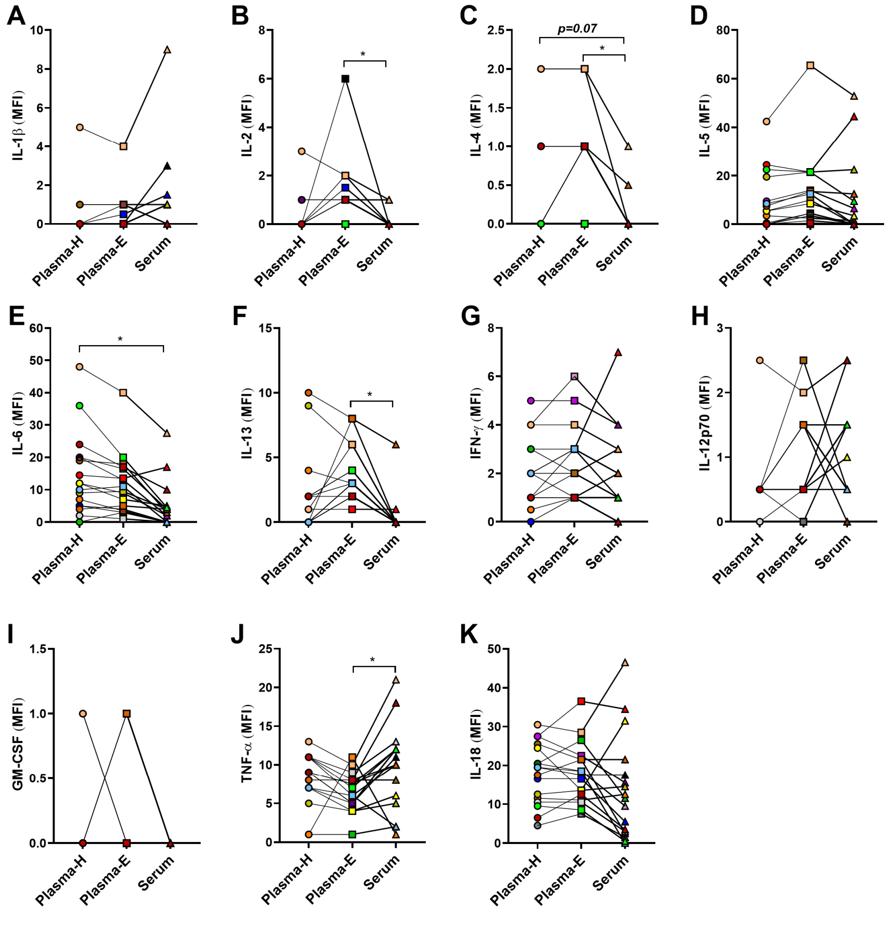

Figure S1: Comparison of cytokine responses (net median fluorescent intensities, Net-MFIs) between plasma and serum samples. Adult (8-10 weeks old) male and female mice were infected either with $10^{1.5}$ TCID$_{50}$ of H1N1 or 10^{2} TCID$_{50}$ of H3N2 IAVs. Mice were euthanized at 3-dpi and blood samples were collected from each mouse to obtain Plasma-H, Plasma-E, and serum samples for the measurement of cytokine responses. Median fluorescent intensities (MFIs) of (A) IL-1β, (B) IL-2, (C) IL-4, (D), IL-5, (E) IL-6, (F) IL-13, (G) IFN-γ, (H) IL-12-p70, (I) GM-CSF, (J) TNF-α, and IL-18 between plasma and serum samples are compared. The same color indicates Plasma-H, Plasma-E, and serum samples from the same animal. Data of 17 animals are shown as symbols and lines and statistical comparison was done using one-way ANOVA followed by Tukey's multiple comparisons test. Asterisk (*) refers to a significant difference at $p<0.05$.

A

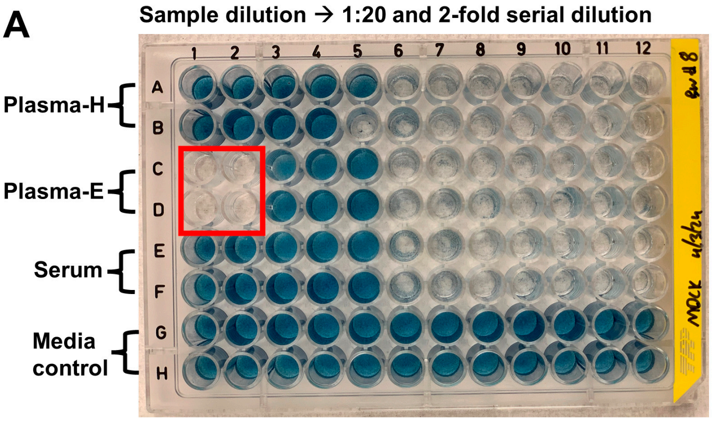

B

		Sample dilution				
**Sample ID**	**Sample type**	10	100	1000	10000	100000
11	Mouse 1 Heparin-Plasma	24515.8	6765.8	82.8	0.0	0.0
12	Mouse 1 EDTA-Plasma	0.0	1223.8	1673.8	0.0	0.0
13	Mouse 1 Serum	38351.8	26254.8	8795.8	119.8	0.0
21	Mouse 2 Heparin-Plasma	29321.8	5867.8	129.8	67.8	0.0
22	Mouse 2 EDTA-Plasma	0.0	0.0	0.0	98.8	0.0
23	Mouse 2 Serum	30021.8	9752.8	190.8	0.0	0.0

Figure S2. Issues observed with Plasma-E in functional antibody assays. (A) A representative image of a plate where Plasma-H, Plasma-E, and serum samples collected from the same mouse are used for neutralizing antibody (nAb) titer measurement in duplicates. The wells (i.e., initial dilutions) having issues in nAb assay while running Plasma-E are highlighted in red. (B) A representative image showing the luminescence readouts in antibody-dependent cellular cytotoxicity (ADCC) assay. Readouts for Plasma-H, Plasma-E, and serum samples of two mice are shown after subtraction of cutoff values (i.e., blank average + 3*standard deviation). Plasma-E had issues in readouts at initial dilutions as highlighted in yellow color.
